# Lymphangioma of the Mesentery: Case Report and Review of the Literature

**DOI:** 10.15388/Amed.2021.28.1.20

**Published:** 2021-05-14

**Authors:** Neda Gendvilaitė, Julius Drachneris, Tomas Poškus

**Affiliations:** Faculty of Medicine, Vilnius University, Vilnius, Lithuania; National Center of Pathology, Affiliate of Vilnius University Hospital Santaros Klinikos, Faculty of Medicine, Vilnius University, Vilnius, Lithuania; Faculty of Medicine, Vilnius University, Vilnius, Lithuania

**Keywords:** lymphangioma, lymphatic system, mesenterium

## Abstract

**Background:**

Lymphangioma of the mesentery is a rare benign condition. Lymphangioma usually occurs in children during first few years of life most likely because of congenital abnormality of the lymphatic system. It may also be caused by trauma, lymphatic obstruction, surgery, inflammatory process, or radiotherapy. Lymphangioma of the mesentery represents less than 1% of all lymphangiomas and about 70% of abdominal lymphangiomas.

**Case presentation:**

We report the case of the 42-year-old woman who was diagnosed with the lymphangioma. Laparotomy was performed. A cystic lymph-filled tumor of about 12 cm in diameter was removed from the ileum mesentery.

**Conclusions:**

Lymphangioma of the mesentery is a rare condition. Despite its benign nature, it can cause serious complications if not treated. Ultrasound and CT are used for detection of lymphangioma. It is important to surgically remove the lymphangiomas even in the absence of symptoms.

## Abstract (lt)

**Apžvalga:**

Plonosios žarnos pasaito limfangiomos yra gerybiniai dariniai. Limfangioma dažniausiai atsiranda vaikams per kelis pirmuosius gyvenimo metus ir paprastai tai lemia įgimtos limfinės sistemos anomalijos. Limfangiomų formavimuisi taip pat turi įtakos traumos, limfinių takų obstrukcijos, chirurginės intervencijos, uždegiminis procesas ar radioterapija. Plonosios žarnos pasaito limfangiomos sudaro mažiau nei 1 % visų limfangiomų ir apie 70 % pilvo ertmės limfangiomų.

**Klinikinis atvejis:**

Pristatomas 42 metų pacientės, kuriai buvo diagnozuota klubinės žarnos pasaito limfangioma, klinikinis atvejis. Buvo atlikta laparotomija. Cistinis limfos pripildytas navikas buvo pašalintas iš klubinės žarnos pasaito.

**Išvados:**

Žarnos pasaito limfangioma yra retas reiškinys. Nors tai yra gerybinis navikas, negydomas jis gali sukelti rimtų komplikacijų. Ultragarsas ir kompiuterinė tomografija yra naudojami limfangiomai aptikti ir nustatyti jos vietą. Svarbu pašalinti limfangiomą, nors klinikinių simptomų ir nebuvo.

**Raktažodžiai:** limfangioma, limfinė sistema, žarnos pasaitas.

## Introduction

Lymphangioma is a benign tumor that usually occurs in children within the first few years of life. It most commonly occurs in the head, neck, and axilla, and these locations carry 95% of all lymphangiomas. The remaining 5% occur in the lungs, mediastinum, adrenal glands, kidney, and bone. Less common places are mesentery, gastrointestinal tract, retroperitoneum, spleen, liver, and pancreas [1]. Childhood lymphangioma represents 80–90% of all lymphangiomas. The reason of lymphangioma mostly occurring in children may be a congenital lymphatic system abnormality and it causes sequestration of the lymphoid tissue during embryonic development. However, it is not completely clear why and how lymphangioma is formed. Mesenteric lymphangioma is rare and it represents less than 1% of all lymphangiomas and 70% of lymphangiomas found in abdominal area [2] [3].

## Case report

**Fig. 1.**
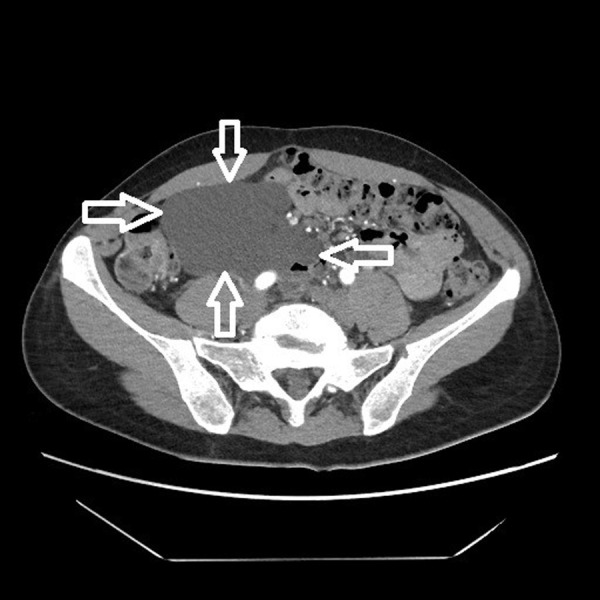
CT scan of the lymphangioma (marked by open arrows) in right abdomen area. The mass is reaching the right side of abdomen wall, contacting abdominal aorta, vena cava and right common iliac artery, also ileocolic artery and vein.

**Fig. 2.**
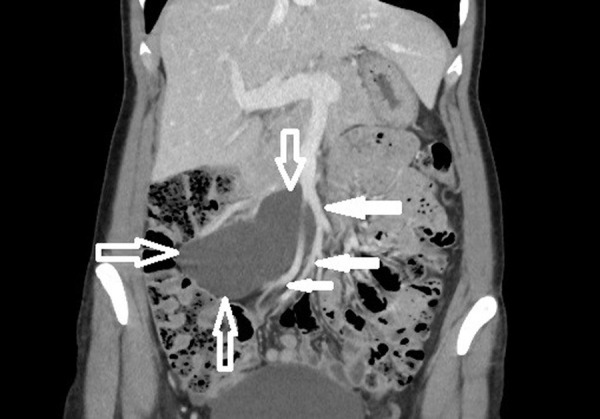
CT scan of the lymphangioma (open arrows) in right abdomen area. The mass extends from pancreatic head and superior mesenteric vein near ileum to right iliac area along the ileocolic artery and vein. Superior mesenteric vein and ileal veins are visible (bold arrows).

The 42-year-old woman sought medical attention for periodically occurring moderately severe pain in the lower abdomen. Intermittent lower abdominal pain occurred 2 years ago for the first time. She was investigated in the emergency department a few times. The pain gradually increased over the time. Physical examination revealed that the patient was well and no physical changes were identified. CT scan revealed a clearly defined, hypodensic, cystic, without clear solid components mass, about 11 cm × 10 cm × 6,5 cm, ventrally reaching the right side of abdomen wall, dorsally contacting abdominal aorta, inferior vena cava and right common iliac artery, caudally lying on the ileocolic artery and vein (Fig. 1 and Fig. 2). The mass extended from pancreatic head and superior mesenteric vein near ileum to right iliac area along the ileocolic artery and vein. Lymphangioma of the ileum mesentery was suspected. Also, an atypical anatomy of the celiac trunk was detected.

**Fig. 3.**
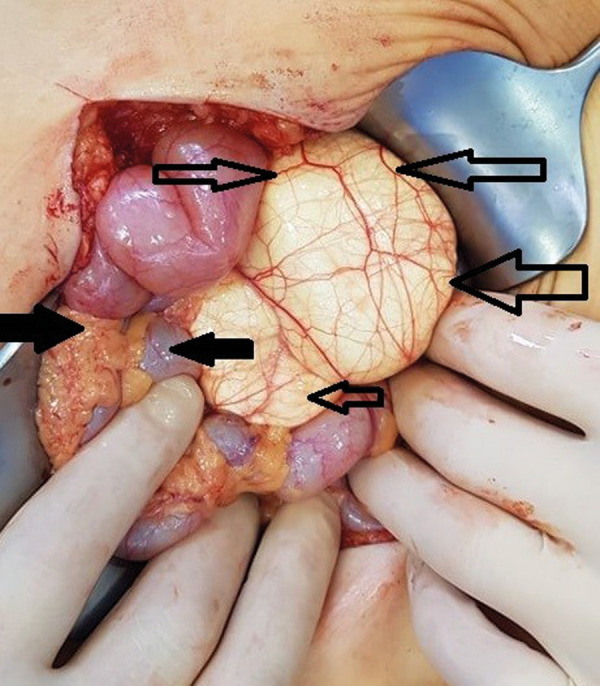
A cystic lymph-filled tumor of about 12 cm in diameter on the ileum mesentery. Open arrows indicate lymphangioma, bold arrows indicate the cecum and the ascending colon

**Fig. 4.**
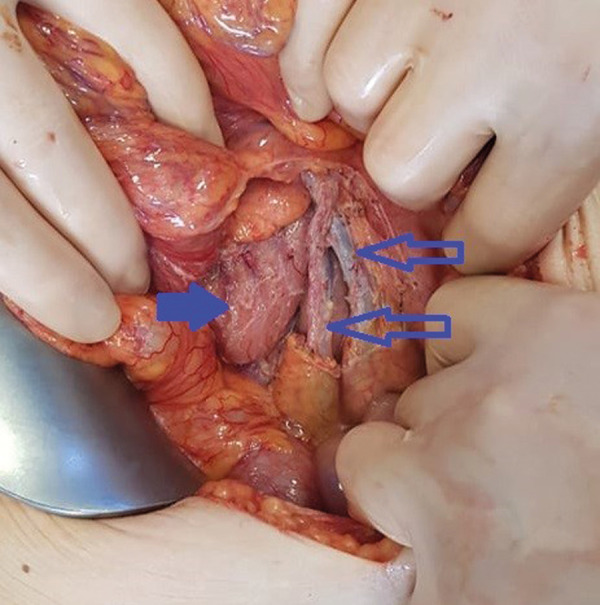
The picture shows the third portion of the duodenum (bold arrow) and the ileocolic and superior mesenteric vessels (open arrows) after the removal of lymphangioma.

After multidisciplinary team discussion the decision was made to proceed with the surgical treatment. Midline laparotomy was performed. A cystic lymph-filled tumor about 12 cm in diameter was detected in the abdominal cavity within the mesentery (Fig. 3). The tumor was dissected from the surrounding structures with vessel sealing device. Right ileocolic artery and vein were freed from the tissues of lymphangioma (Fig. 4). No extravasation of the lymph flow was observed after removal of the mass. 

**Fig. 5.**
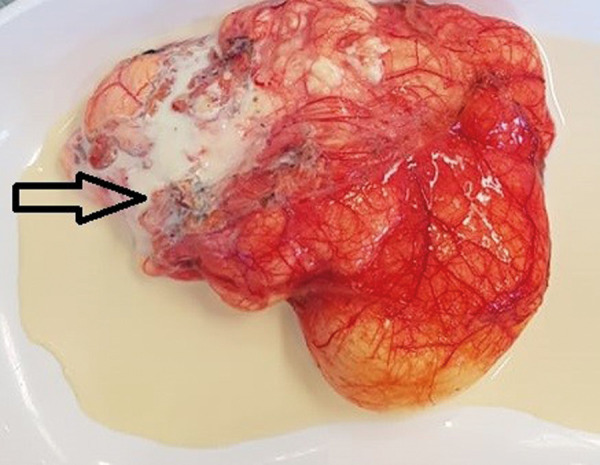
The picture shows milk-like fluid (open arrow) coming out of the lymphangioma. The fluid color is similar to milk due to the accumulation of lymphocytes.

**Fig. 6.**
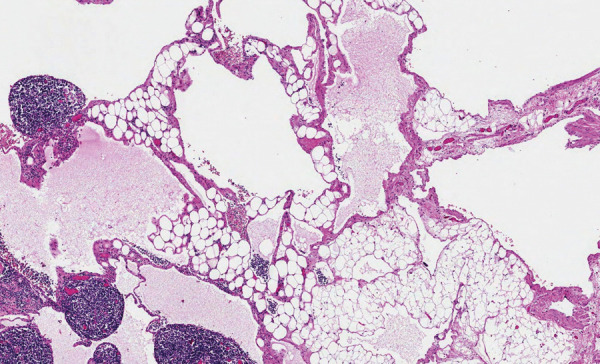
Cystic tumor structures with lymphoid aggregates. 40x magnification hematoxylin and eosin.

**Fig. 7.**
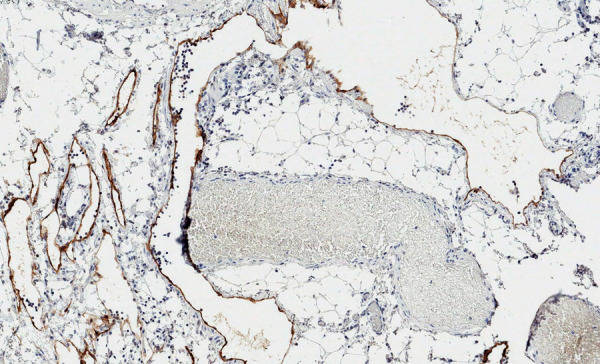
Lining of tumor structures positive for podoplanin (D2-40). 100x magnification.

On pathological examination, the macroscopically observed partially opened fatty-fibrous cystic formation 9 cm × 5 cm × 3 cm was identified. Cysts of up to 2 cm filled with whitish, turbid substance were visible. 6 lymph nodes up to 0.5 cm were excised alongside with the lymphangioma. Histologically thin-walled cystic tumor structures were separated by areas of adipose tissue with lymphoid aggregates (Fig. 6). The cavities of the mass were filled with eosinophilic fluid and lymphocytes. The fluid color is similar to milk due to the accumulation of lymphocytes (Fig. 5). Lymphoid aggregates and lymph nodes with histiocytosis were found in the surrounding tissue. Immunohistochemical examination of the lymphatic endothelial marker podoplanin (D2-40) was positive in the endothelial wall of the cystic tumor (Fig. 7). Histological findings support the diagnosis of lymphangioma.

## Discussion

Lymphangioma is a benign condition that results from the proliferation of lymphatic cavities with thinned walls [2]. About 95% of lymphangiomas occur in the head, neck or axilla. The remaining 5% are found in the lungs, mediastinum, adrenal glands, kidney, bone. Less common places are mesentery, gastrointestinal tract, retroperitoneum, spleen, liver, and pancreas [1]. Lymphangiomas are most common in children. 80–90% of all lymphangiomas occur within first few years of life. Lymphangioma of the small bowel mesentery is rare, especially in adults. It represents less than 1% of all lymphangiomas and about 70% of abdominal lymphangiomas [2] [3].

The etiology of lymphangioma is not entirely clear. They are thought to result from a congenital lymphatic system abnormality that causes sequestration of the lymphoid tissue during embryonic development. This theory would substantiate why lymphangiomas are most commonly seen in children. Also, there are theories that secondary lymphangioma formation is provoked by abdominal trauma, lymphatic obstruction, surgery, inflammatory process, or radiotherapy [2] [3].

There are three types of lymphangiomas: simple (capillary), cavernous, and cystic. Simple lymphangioma occurs in the superficial layer of the skin and is made of small thin-walled lymphatic spaces. Cavernous lymphangioma consists of dilatated lymphatic vessels and lymphoid stroma and connects with spaces of adjacent normal lymphatics. Cystic lymphangioma consists of lymphatic spaces of various sizes that contains smooth muscle fibers and collagen in the stroma but have no connections to adjacent normal lymphatic spaces. The cystic type cannot always be clearly distinguished from the cavernous type because a cystic lymphangioma may have cavernous areas [4].

Bowel mesentery lymphangioma is usually asymptomatic until it increases in size sufficiently. The most common symptoms are abdominal pain and increased abdominal volume. However, clinical manifestation may vary. Although mesenteric lymphangioma is a benign tumor, its size and location may lead to morbidity when other adjacent organs and structures are affected. Complications such as secondary infection, rupture with bleeding, and intestinal obstruction may occur [3].

Ultrasound examination of mesenteric lymphangioma usually reveals a cystic multiseptated mass. CT scan shows uni- or multilocular mass with a contrast-accumulating capsule and septa [5]. These studies help to determine the location, size, and type of lymphangioma. However, ultrasound and CT scans are not enough to settle the exact diagnosis prior to surgical treatment. There are some cases where aspiration of a milk-like fluid from lymphangioma may confirm the diagnosis [3].

Mesenteric lymphangiomas may grow to a very large size and invade the surrounding cavities and organs. The treatment is to surgically remove lymphangioma, even if no symptoms have occurred yet. This treatment may be complicated due the penetration of lymphangioma to other structures such as the main mesenteric artery or intestine. In that case segmental bowel resection may be performed. Sometimes radical resections may not be possible [6].

## Conclusion

The small bowel mesentery lymphangioma is a rare condition that can cause serious complications. Lymphangioma of any kind occurs most often in children because of congenital anomalies of the lymphatic system. Cases in adults represent only 10–20%. They can be detected by the ultrasound or CT scans and confirmed via aspiration of milk-like fluid or histological findings. Although lymphangiomas may be asymptomatic, if detected, they have to be surgically removed. 
